# MnSOD Mimetics in Therapy: Exploring Their Role in Combating Oxidative Stress-Related Diseases

**DOI:** 10.3390/antiox13121444

**Published:** 2024-11-23

**Authors:** Jovan Grujicic, Antiño R. Allen

**Affiliations:** 1 Division of Radiation Health, University of Arkansas for Medical Sciences, Little Rock, AR 72205, USA; jgrujicic@uams.edu; 2 Department of Pharmacology and Toxicology, University of Arkansas for Medical Sciences, Little Rock, AR 72205, USA; 3 Department of Pharmaceutical Sciences, University of Arkansas for Medical Sciences, Little Rock, AR 72205, USA; 4 Neurobiology and Developmental Sciences, University of Arkansas for Medical Sciences, Little Rock, AR 72205, USA

**Keywords:** manganese superoxide dismutase (MnSOD), MnSOD mimetics, oxidative stress, reactive oxygen species (ROS), Mn porphyrins, Mn salens, MitoQ10, nitroxides, mangafodipir, mitochondria-targeted antioxidants

## Abstract

Reactive oxygen species (ROS) are double-edged swords in biological systems—they are essential for normal cellular functions but can cause damage when accumulated due to oxidative stress. Manganese superoxide dismutase (MnSOD), located in the mitochondrial matrix, is a key enzyme that neutralizes superoxide radicals (O_2_^•−^), maintaining cellular redox balance and integrity. This review examines the development and therapeutic potential of MnSOD mimetics—synthetic compounds designed to replicate MnSOD’s antioxidant activity. We focus on five main types: Mn porphyrins, Mn salens, MitoQ10, nitroxides, and mangafodipir. These mimetics have shown promise in treating a range of oxidative stress-related conditions, including cardiovascular diseases, neurodegenerative disorders, cancer, and metabolic syndromes. By emulating natural antioxidant defenses, MnSOD mimetics offer innovative strategies to combat diseases linked to mitochondrial dysfunction and ROS accumulation. Future research should aim to optimize these compounds for better stability, bioavailability, and safety, paving the way for their translation into effective clinical therapies.

## 1. Introduction

Controlled redox reactions occur regularly within various biological processes, such as cellular respiration, signal transduction, gene expression, and maintenance of cellular homeostasis [1]. Redox balance, the equilibrium between oxidation and reduction reactions in the cell, is vital for the continued health of an organism. Its disruption leads to oxidative stress and the overaccumulation of reactive oxygen species (ROS). Overaccumulated ROS can react with lipids, proteins, and nucleic acids, damaging them in the process and compromising cellular integrity [2].

Superoxide dismutase (SOD) isoforms play a critical role in protecting cells from oxidative stress by neutralizing O_2_^•−^ [1]. Manganese SOD (MnSOD), exclusively located within the mitochondrial matrix and the only SOD in the mitochondria, is a critical enzyme for maintaining mitochondrial function by reducing ROS levels, preventing mitochondrial damage, and managing oxidative stress in this organelle, which is estimated to produce 90% of cellular ROS [3,4,5]. It plays the most significant role in tissues with high metabolic demand, where its expression is the highest [6].

The deletion or alteration of MnSOD impacts obesity, metabolic health, and oxidative stress. Studies indicate that in high-fat diets, adipocyte-specific MnSOD gene knockout mice exhibit reduced adiposity and improved glucose tolerance, highlighting MnSOD’s therapeutic potential in metabolic regulation [7]. Genetic polymorphisms in MnSOD, such as Ala16Val, substitution at position 16 of the MnSOD protein, and changing an alanine (Ala) to a valine (Val), are associated with obesity, where the Val variant disrupts mitochondrial targeting and links MnSOD dysfunction with increased obesity risk [8]. Similarly, MnSOD deficiency in enterocytes promotes obesity via arachidonic acid-driven inflammation, supporting the enzyme’s role in combating obesity [9]. In obese pregnant women, reduced MnSOD and mitochondrial enzyme activity emphasize the oxidative stress implications of obesity beyond gestational diabetes [10]. MnSOD also plays a protective role in kidney health, mitigating oxidative damage in chronic kidney disease and acute kidney injury, where its deficiency accelerates renal inflammation and damage, underscoring its importance in renal pathophysiology [11,12]. In diabetes, MnSOD limits ROS and improves islet function, reducing complications such as nephropathy and retinopathy [13], with the Ala16Val polymorphism further modulating diabetic nephropathy risk across populations and linking genetic variations to differential disease susceptibility [14]. In cardiovascular health, MnSOD is essential for managing oxidative stress and reducing risks associated with hypertension and atherosclerosis, as MnSOD deficiency in cardiomyocytes can lead to heart failure, highlighting the enzyme’s vital role in preserving mitochondrial and cardiac function [15,16]. Additionally, MnSOD polymorphisms are linked to elevated coronary artery disease risk, and therapeutic upregulation of MnSOD could offer protective effects against cardiovascular conditions [15]. In cancer, decreased MnSOD expression is linked to mechanisms such as epigenetic silencing, promoter methylation, mutations, and loss of heterozygosity, as seen in cancers like brain, breast, colorectal, and pancreatic [17]. Post-translational modifications, including hyperacetylation, nitration, and oxidation, are linked to reduced MnSOD activity, as evident in renal cancer, glioma, and certain B-cell malignancies [17]. In aggressive cancer types, overexpressed MnSOD correlates with worse outcomes, suggesting that therapeutic targeting of MnSOD pathways may improve treatment effectiveness [18]. In neurological health, MnSOD protects against oxidative damage in conditions like epilepsy, Huntington’s, and Parkinson’s, where lower MnSOD levels correlate with exacerbated symptoms, while MnSOD mimetics show therapeutic promise in alleviating neurodegenerative disease outcomes [19,20]. Due to the profound connections between altered MnSOD states and severe health disorders across various organ systems, MnSOD mimetics represent promising treatment options in conditions where MnSOD is implicated. Similarly, due to their ability to regulate oxidative stress, MnSOD mimetics are also promising treatment options in a wider context than supplementing MnSOD deficiency/malfunction.

Human MnSOD forms a homotetramer consisting of four identical subunits, each containing an active site with a manganese ion as the cofactor. The tetrameric structure provides the enzyme stability and improves the dimer-dimer interactions, consequently improving function [21]. A pulse-radiolysis study in 1977 by McAdam et al. observed that at low substrate-to-enzyme ratios, superoxide decay is nearly exponential, whereas, at high ratios, it becomes zero-order, indicating a rapid oxidation-reduction cycle and a slower process that regenerates the active enzyme from an inactive form [22]. This allowed them to distinguish two distinct pathways totaling four reactions (Table 1) that enable MnSOD catalytic activity: (1) the fast outer sphere pathway, where superoxide is directly converted to hydrogen peroxide (H_2_O_2_), and (2) the slow inner sphere pathway, which involves the formation of a product-inhibited complex followed by the release of H_2_O_2_ [22]. In both pathways, superoxide binds directly to manganese at rate constant k_1_, reducing it from 3+ to 2+ and using Gln142 as the protonation source to protonate the hydroxide ligand to H_2_O [22,23] (Table 1, Full Equation (1)). In the fast pathway, at a rate constant k_2_, a second superoxide bonds with the ligand water and Tyr34, converting superoxide to H_2_O_2_ while oxidizing Mn^2+^ to Mn^3+^ and regenerating the resting state of the enzyme (Table 1, Full Equation (2)) [22,24]. In the slow pathway, which occurs at high superoxide concentrations and low temperature, a second superoxide forms a reversible peroxide adduct with Mn^2+^ at rate constant k_3_, inhibiting the enzyme; after a period of inactivity, two protons from the water ligand and outer sphere network form H_2_O_2_ and are released, oxidizing the manganese back to its resting Mn^3+^ state bound to the hydroxyl ligand, where the protonation rate is equal to rate constant k_4_ (Table 1, Full Equation (4)) [25].

Historically, after Harman’s theory of aging from the mid-50s and the fact that oxidative stress can cause significant damage to the body became widely accepted, ways of combating oxidative stress became a popular field of study [7]. Initially, scientists were interested in understanding SODs in general. At the core of the SOD mechanism is the dismutation of the O_2_^•−^ into molecular oxygen (O_2_) and H_2_O_2_.

Scientists wanted to develop mimetics, synthetic enzymes capable of mimicking MnSODs catalytic mechanism. In the 1970s, this led to the creation of Fe porphyrin [13]. As SODs mechanistically rely on redox-active metal sites, the first SOD mimics explored were metal complexes. Due to their redox activity and stability provided by the porphyrin ligand, iron porphyrins were the first studied. Free iron has a high risk of causing toxicity via the Fenton reaction, which leads to favoring Mn porphyrins, which are now classified as a group within MnSOD mimetics [13].

Mn salen derivatives, cyclic polyamines, corroles, and non-metal compounds like nitroxides and nitrones were synthesized and studied next, all capable of mimicking MnSOD [13]. It was only in the early 2010s that the focus of the research on these compounds changed from their general SOD properties to their ability to specifically mimic MnSOD [13]. Some of these compounds, like Mn cyclic polyamines and redox-active corroles, were found unsuitable due to their lack of positive charge required for mitochondrial targeting [14,15]. At the same time, interest in MitoQ compounds, specifically MitoQ 10, arose as it had a promising ability to accumulate in the mitochondria due to its positive charge [16,17,18]. However, Mn porphyrins were not given up on, as conjugating mitochondria-targeted sequences to them had shown promise [19].

The main types of MnSOD studied today, which will be discussed, are Mn porphyrins, Mn salens, mitoQ 10, nitroxides, and mangafodipir. It is important to note that some MnSOD mimetics, like Mn porphyrins, Mn salens, and mangafodipir, are designed to replicate MnSOD’s mechanism, aiming to emulate the catalytic activity of MnSOD by facilitating similar redox cycles involving manganese ions. These mimetics typically incorporate manganese to support rapid superoxide conversion, as seen in MnSOD’s distinct fast and slow pathways. In contrast, compounds like MitoQ10 and Nitroxides, which lack manganese, are categorized as MnSOD mimetics due to their targeted superoxide neutralization rather than mechanistic mimicry. This review will focus on research on potential therapeutic applications of MnSOD mimetics. Integrating contemporary research insights and methods, we aim to present a cohesive and critical evaluation of these agents in a broad context.

## 2. MnSOD Mimetics

### 2.1. Mn Porphyrins

**Figure 1:**
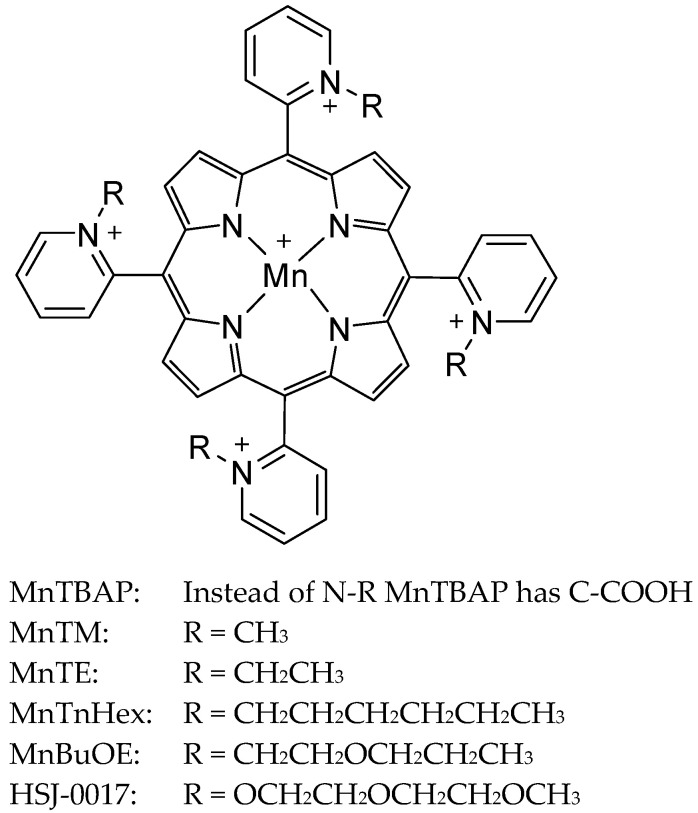
Core structure of Mn Porphyrins.

Mn porphyrins are a class of synthetic compounds designed to mimic the activity of MnSOD. These compounds consist of a manganese ion centrally coordinated within a porphyrin ring, a large, stable, cyclic molecule that can bind metal ions (Figure 1). The reduction potential of the metal site in enzymes is carefully balanced to optimize the catalytic dismutation of superoxide [26]. By modifying the structure of Mn porphyrins with electron-withdrawing groups, researchers increased their electron deficiency, which enhanced the reduction potential, leading to better mimicry of the natural enzyme’s activity [26]. However, while these modifications improved catalytic efficiency, they also introduced challenges, such as stability issues under physiological conditions and potential toxicity due to micellar properties. To address these, efforts focused on balancing lipophilicity and charge distribution, ultimately developing more effective and safer Mn porphyrin compounds with therapeutic potential across various conditions [26]. Mn porphyrins localize well within the mitochondria as they possess a pentacationic charge and five positive charges (cationic charge plays a critical role in mitochondrial drug localization) [27]. Additionally, it is well-documented that increased lipophilicity increases mitochondrial accumulation, blood–brain barrier crossing, and tumor localization [28,29,30,31,32]. Mn porphyrins modulate cellular redox balance by influencing reactive species levels and activating transcription factors, effectively reducing oxidative stress and inflammation through interactions with proteins like nuclear factor kappa-light-chain-enhancer of activated B cells [26]. Their reactivity with cellular reductants such as ascorbate and glutathione highlights their potential as therapeutic agents for managing oxidative stress-related conditions [26]. There are many therapeutically tested Mn porphyrins with varied structures (Figure 1, Table 2).

MnTBAP is a manganese porphyrin-based compound where the central manganese ion is surrounded by a tetrakis (4-benzoic acid) porphyrin ring, which enhances its stability and redox activity. Due to this structure, MnTBAP was initially investigated as a potential SOD mimetic. However, its identity has become a matter of debate as subsequent studies revealed that MnTBAP does not effectively function as a SOD mimetic in aqueous systems due to its unfavorable reduction potential for superoxide dismutation [61]. MnTBAP primarily acts as a broad-spectrum antioxidant. It scavenges ROS like H_2_O_2_ and peroxynitrite, showing a significantly higher preference for these mechanisms than superoxide dismutation [62]. Despite this, by design, MnTBAP is one of the earliest MnSOD mimetics developed, and it has continuously been used in research on various diseases, warranting its inclusion in this review. Due to its age and broad antioxidant effects, MnTBAP has been investigated in hundreds of papers, making it necessary to be more selective in review.

Within the past four years, MnTBAP has demonstrated beneficial effects in reproductive biology. It has shown significant potential as a protective agent in sperm cryopreservation across various studies [33,34,35,36]. This antioxidant mimetic effectively lowers ROS levels, enhances sperm viability, and maintains motility, which is crucial for successful sperm preservation [33,35]. In the vitrification process, MnTBAP was particularly beneficial when added during the warming and post-warming incubation steps, where it preserved membrane integrity and decreased ROS production without compromising sperm functionality [35]. In cryopreservation of human sperm, MnTBAP further proved its efficacy by reducing sperm DNA fragmentation, apoptosis, and structural protein damage, such as A-kinase anchoring protein 4, which is essential for sperm motility [36]. The use of MnTBAP significantly improved motility and minimized proteomic alterations, resulting in enhanced sperm survival after thawing [36]. Most recently, in porcine semen cryopreservation, MnTBAP demonstrated a pronounced effect on motility, acrosome integrity, and mitochondrial membrane potential, leading to higher embryo development rates in vitro [34]. Together, these studies indicate that MnTBAP offers substantial benefits in preserving sperm quality across different cryopreservation methods, making it a promising candidate for further development in reproductive medicine.

Recent studies involving MnTBAP revealed potential in mitigating vascular disorders [37,38]. By increasing bone morphogenetic protein receptor type 2 levels and inhibiting autophagy in pulmonary endothelial and smooth muscle cells, MnTBAP significantly reduced right ventricular (RV) afterload and reversed pulmonary vascular remodeling in a rat model of pulmonary arterial hypertension [37]. In a separate study, this mimetic demonstrated efficacy in reducing vascular oxidative stress and preventing vascular remodeling in models of SIRT2 deficiency, suppressing oxidative damage to proteins, lipids, and DNA [38]. This reduction in oxidative stress also prevented arterial stiffening by inhibiting matrix metalloproteinases, key drivers of collagen deposition and arterial stiffness [38].

Lastly, three recent studies have provided insights into MnTBAP’s ability to mitigate the toxic side effects of cisplatin chemotherapy [28,39,40]. In a cisplatin-induced acute kidney injury model, MnTBAP acted as a mitochondrial ROS scavenger, effectively reducing mitochondrial ROS levels and restoring mitochondrial homeostasis [39]. This reduction in oxidative stress was critical in alleviating ferroptosis, a form of cell death triggered by lipid peroxidation, which plays a key role in renal tubular injury [39]. Furthermore, MnTBAP helped preserve mitochondrial structure and function by maintaining the oxidative phosphorylation system, thereby preventing further kidney damage [28]. In another study, MnTBAP was combined with NSC228155 (NSC228155 enhances epidermal growth factor receptor activation and inhibits cyclic AMP response element-binding protein (creb) and creb-binding protein interaction, impacting cell proliferation and transcription), where it further demonstrated its protective effects by reducing tubular injury, serum creatinine, and blood urea nitrogen levels in models of cisplatin-induced renal damage [39]. The overlapping mechanisms of both compounds highlight MnTBAP’s role in mitigating oxidative and endoplasmic reticulum (ER) stress, which are major contributors to cisplatin-induced nephrotoxicity [38]. Beyond kidney protection, MnTBAP has also been shown to prevent cisplatin-induced hearing loss by reducing nitrative stress in cochlear synapses [40]. MnTBAP preserved synaptic integrity and auditory function by scavenging peroxynitrite, a reactive nitrogen species, and preventing the nitration of synaptic proteins, which are crucial for proper signal transmission in the auditory pathway [40].

MnTM-2-PyP (MnTM) is a manganese porphyrin characterized by a tetrakis(N-methylpyridinium-2-yl) porphyrin ring, which imparts significant redox properties and effectively mimics the activity of the natural SOD enzyme. This structure enhances MnTM’s ability to catalyze the dismutation of superoxide anions. However, it exhibits lower cellular uptake than its later-developed, more lipophilic counterparts. Due to this, the last disease-specific research that focused on MnTM was conducted in 2013, when its impact on diabetic complications was evaluated. Delayed administration of MnTM, eight days post-diabetes onset, exacerbated kidney damage rather than providing protection, indicating that timing is critical for its therapeutic efficacy. This study highlighted the importance of early intervention with manganese porphyrin compounds to avoid worsening oxidative damage under conditions of advanced oxidative stress [41]. Since then, research has shifted toward more advanced MnSOD mimetics that offer greater cellular uptake and therapeutic potential.

MnTE-2-PyP5+ (MnTE) is a highly efficient manganese porphyrin with a tetrakis(N-ethylpyridinium-2-yl) porphyrin structure, giving it potent antioxidant properties and strong SOD mimetic activity. While research on MnTE has slowed in favor of newer alternatives, it could still serve as a valuable benchmark for evaluating novel therapeutics. Despite its reduced prominence, several findings have emerged within the past decade. MnTE has shown significant potential in reversing and preventing acute vaso-occlusive crises in a sickle cell disease model [46]. This manganese porphyrin reduces the adhesion of sickle red blood cells and leukocytes, decreases oxidative stress by inhibiting NADPH oxidases, and improves blood flow and survival rates in sickle mice [46]. The treatment was well-tolerated and substantially reduced oxidative stress markers and leukocytosis [46]. Nitric oxide donor S-nitrosoglutathione (GSNO) is known for its cardioprotective properties in healthy animals. A study found that while GSNO significantly reduced infarct size and improved heart function in non-diabetic mice, it exacerbated myocardial ischemia/reperfusion (MI/R) injury in diabetic mice, leading to increased infarct size and worsened cardiac function [44]. Co-administration with MnTE mitigated these harmful effects, reducing peroxynitrite production and limiting MI/R injury [44]. Additionally, MnTE was shown to modulate immune cell metabolism in type 1 diabetes by altering metabolic pathways in diabetogenic splenocytes, reducing immune cell activation and proliferation, and potentially offering a therapeutic strategy for autoimmune diseases [43].

In radiation-induced proctitis, MnTE effectively mitigated both acute and chronic symptoms when administered before irradiation, suggesting its utility in reducing gastrointestinal toxicity during radiotherapy. However, post-treatment proved less effective, highlighting the importance of timing in its application [42]. Additionally, MnTE has been shown to significantly enhance tumor suppression when combined with radiation therapy and ascorbate. This redox-active compound cycles with ascorbate to generate H_2_O_2_, which induces protein S-glutathionylation, mimicking the activity of glutathione peroxidase. This process shifts the cellular redox balance by increasing the glutathione disulfide/glutathione ratio, ultimately leading to greater tumor growth inhibition [45].

MnTnHex-2-PyP5+ (MnTnHex) is a lipophilic manganese porphyrin with hexyl side chains integrated into its porphyrin ring, enhancing its membrane permeability and tissue accumulation. This structural modification allows MnTnHex to penetrate cells more effectively and localize within organelles such as mitochondria, where oxidative damage is often most pronounced. In mice, MnTnHex demonstrated high efficacy as a radioprotective agent in reducing radiation-induced lung damage, including oxidative stress and fibrosis, at significantly lower doses compared to its hydrophilic counterpart, MnTE. However, caution is warranted due to its surfactant-like properties, which could lead to toxicity at higher doses [50]. This was also demonstrated in primates, where MnTnHex reduced lung injury by lowering respiratory rates, delaying the onset of lung lesions, and reducing pathological increases in lung weight in irradiated non-human primates [52]. Moreover, MnTnHex was shown to reverse PTEN promoter (promoter for phosphatase and tensin homolog gene) hypomethylation in radiation-induced lung injury, indicating its potential to mitigate epigenetic changes associated with oxidative stress [51]. Recently, MnTnHex has been mostly studied in cancer therapy [47,48,49]. It enhanced radiation-induced cell death in murine mammary carcinoma and melanoma cells by increasing oxidative stress within tumors while protecting normal tissues, thereby broadening the therapeutic window in radiotherapy [47]. In non-small cell lung cancer cell lines, MnTnHex exhibited significant cytotoxicity at sub-micromolar concentrations, disrupting cell viability and cycle distribution [48]. When used in combination with cisplatin, MnTnHex enhanced the efficacy of the chemotherapeutic’s efficacy, reducing cell migration and increasing cell death [48]. MnTnHex was also found to significantly reduce the viability and chemotactic migration of 786-O human renal cancer cells, most likely due to the redox activity of MnTnHex, leading to the production of H_2_O_2_, which cancer cells, particularly renal cancer cells, are less capable of detoxifying [49].

MnTnBuOE-2-PyP5+ (MnBuOE) is a manganese porphyrin designed with butoxy ethyl side chains, significantly improving its lipophilicity and bioavailability. This structural enhancement allows MnBuOE to efficiently cross cell membranes and accumulate in tissues, making it highly effective in targeting oxidative stress within both intracellular and extracellular environments. MnBuOE emerged as a versatile therapeutic agent across various conditions [53,54,55,56,57,58,59]. It provided significant protection against radiation-induced normal tissue injury, including the preservation of hippocampal neurogenesis and reduction in cognitive deficits following cranial irradiation. This neuroprotective effect is particularly relevant in preserving cognitive function during central nervous system-targeted cancer treatments [53]. Additionally, MnBuOE enhanced the effectiveness of cancer therapies in glioblastoma, ovarian cancer, and non-small cell lung cancer by increasing oxidative stress selectively within cancer cells, thereby sensitizing them to chemotherapeutic agents while protecting normal tissues [54,55,56,57]. MnBuOE also showed promise in improving outcomes following cardiac arrest by enhancing survival rates and reducing organ damage [58]. MnBuOE also demonstrated effectiveness in managing hypertension. In hypertensive animal models, MnBuOE significantly lowered elevated blood pressure through sympathoinhibition and vasodilation, partially mediated by nitric oxide [59]. These effects were dose-dependent, with higher doses prolonging the hypotensive response.

HSJ-0017 is a manganese porphyrin compound that has been engineered to provide dual antioxidant protection by mimicking the activities of both SOD and catalase. This unique combination gives HSJ-0017 broader utility in reducing oxidative damage. There hasn’t been much research utilizing this compound yet. HSJ-0017 demonstrated significant antioxidant activity in vitro, inhibiting the generation of superoxide anions and scavenging (H_2_O_2_) in a dose-dependent manner [60]. in vivo, it had notable antitumor effects in sarcoma 180 tumor-bearing mice but was less effective against hepatocarcinoma 22 tumor xenografts [60]. HSJ-0017 also enhanced the effects of chemotherapy and radiotherapy while mitigating their toxic side effects [60]. Additionally, the compound showed significant anti-inflammatory and hepatoprotective effects, reducing oxidative damage in models of carbon tetrachloride-induced hepatic injury [60].

Mn porphyrins have emerged as versatile therapeutic agents due to their ability to mimic the enzyme MnSOD. These compounds have been extensively modified to enhance their electron deficiency, thereby improving their catalytic efficiency for superoxide dismutation. However, these modifications have introduced challenges such as stability issues and potential toxicity. Efforts to address these limitations, particularly by balancing lipophilicity and charge distribution, have led to the development of more effective and safer Mn porphyrins with therapeutic applications across various conditions. Mn porphyrins are especially well-suited for mitochondrial targeting due to their cationic charge and lipophilicity, which enhance mitochondrial and tissue accumulation. Their ability to modulate cellular redox balance by scavenging ROS and interacting with cellular reductants highlights their potential in managing oxidative stress and inflammation. MnTBAP, one of the earliest MnSOD mimetics, primarily acts as a broad-spectrum antioxidant, showing promise in sperm cryopreservation and mitigating oxidative damage in vascular and kidney models. MnTM has seen limited recent use due to its lower cellular uptake, with studies emphasizing the need for early intervention to prevent exacerbated oxidative stress. MnTE remains an important benchmark in redox research, demonstrating efficacy in enhancing radiation therapy and mitigating autoimmune conditions by selectively targeting oxidative stress. MnTnHex enhances tissue penetration and shows significant promise in cancer and radioprotection, increasing oxidative stress within tumors while protecting healthy tissue. MnBuOE has shown versatility in protecting against radiation-induced cognitive deficits, improving outcomes in cancer therapies, and reducing hypertension via sympathoinhibition. Lastly, HSJ-0017, a novel Mn porphyrin with dual SOD and catalase mimetic activities, has demonstrated antioxidant, anti-inflammatory, and hepatoprotective effects, though further research is needed. Collectively, Mn porphyrins continue to evolve as potent therapeutic agents across various oxidative stress-related conditions.

### 2.2. Mn Salens

Mn Salens are a group of synthetic compounds that have gained significant attention due to their potential as SOD mimics, particularly for their ability to manage oxidative stress in various biological systems. Structurally, Mn salens are characterized by the presence of a manganese ion coordinated with a salen ligand, which is formed from the condensation of salicylaldehyde and ethylenediamine. This configuration allows Mn salens to catalyze the dismutation of O_2_^•−^ into less harmful species like H_2_O_2_ and O_2_, thereby mimicking the natural activity of SOD enzymes. The main Chemotherapeutically tested Mn salens are EUK-134, EUK-207 and EUK-8.

EUK-134 is a synthetic catalytic antioxidant mimicking SOD and catalase that has been studied in many diseases. It was found effective in reducing hyperoxic stress-induced activation of the transforming growth factor-beta 1 (TGF-β1)/Smad2 signaling pathway in myocardial tissue following ischemia and reperfusion injury [63]. The treatment blunted the increase in Smad2 phosphorylation, which is associated with fibroblast trans-differentiation into myofibroblasts, suggesting a protective role in managing post-ischemic myocardial remodeling by modulating oxidative stress and its downstream effects on TGF-β1 signaling [63].

EUK-134 demonstrated significant potential as a therapeutic agent in breast cancer treatment by reducing superoxide and H_2_O_2_ levels within breast cancer cells, leading to decreased proliferation and viability of the cell lines MCF-7 (human metastatic breast cancer) and MDA-MB-231 (human triple-negative breast cancer) [64]. The compound induced cell cycle arrest in the G2-M phase and triggered apoptosis, while also markedly inhibiting cancer cell migration and adhesion [64]. These findings suggest EUK-134’s promising role in targeting oxidative stress and inhibiting critical aspects of cancer progression [64].

In the context of skeletal muscle subjected to short-term mechanical unloading, EUK-134 significantly protected against muscle fiber atrophy and prevented the fiber-type shift from slow-twitch (Type I) to fast-twitch (Type II). The compound also inhibited the translocation of neuronal nitric oxide synthase from the sarcolemma to the cytosol, which is linked to muscle atrophy, and mitigated oxidative stress by normalizing levels of Nox2, a subunit of NADPH oxidase associated with oxidative damage [65,66]. This highlights EUK-134’s potential as a protective agent against muscle morphology deterioration during disuse [65,66].

EUK-134 was shown to inhibit platelet aggregation induced by thromboxane A2 analogs, particularly when combined with epinephrine [67]. This suggests that EUK-134 is effective in reducing ROS generation, which is crucial for platelet activation, indicating its potential role in managing platelet-related conditions [67].

A study on ER stress and mitochondrial dysfunction found that EUK-134 could mitigate several detrimental effects caused by ER stress, including the loss of mitochondrial membrane potential and respiratory dysfunction in human skeletal muscle cells [68]. This suggests that EUK-134 has the potential to protect against muscle weakness and dysfunction related to chronic ER stress by targeting ROS generation, making it a promising therapeutic option for conditions like myositis [68].

When tested in non-alcoholic steatohepatitis (NASH), EUK-134 was found to effectively reduce serum levels of liver damage markers and improve pathological features in rats fed a methionine/choline-deficient diet [69,70]. The compound’s ability to prevent lipid peroxidation and protein carbonyl formation suggests its potential as a therapeutic agent for NASH by mitigating oxidative stress and liver damage [69,70].

EUK-134 effectively prevented diaphragm muscle weakness in a rat model of monocrotaline-induced pulmonary hypertension by protecting against the PH-induced decrease in specific diaphragm force and preventing oxidative stress-induced modifications of myofibrillar proteins [71]. This suggests EUK-134’s potential as a therapeutic strategy to counteract muscle weakness in conditions associated with oxidative stress [71].

EUK-207 has been studied less but has still shown promising effects. A study highlighted the efficacy of EUK-207 in reducing radiation-induced lung damage, specifically in delaying and decreasing the severity of pneumonitis in adolescent rats [72,73]. EUK-207 lowered oxidative damage and inflammation markers, providing substantial protection against radiation-induced lung injury even when treatment was initiated weeks after exposure [72,73]. This positions EUK-207 as a potential radioprotective agent, particularly when immediate treatment is not feasible [72,73].

EUK-207 significantly mitigated radiation dermatitis and promoted wound healing in irradiated rat skin [74]. Administered systemically after irradiation, EUK-207 reduced the severity of skin injury and accelerated wound healing by normalizing gene expression related to oxidative stress and reducing oxidative damage to proteins and DNA [74]. These findings support its potential as a therapeutic agent for treating radiation-induced skin injuries [74].

EUK-207 also mitigated radiation-induced cognitive impairments in mice, particularly in reducing hippocampus-dependent spatial memory deficits caused by high-dose radiation [75]. The compound’s ability to prevent oxidative damage without negatively impacting cognitive performance in sham-irradiated mice suggests its promise of protecting against radiation-induced cognitive injury [75].

Similarly, EUK-8 more therapeutic applications of EUK-8 should be studied; however, so far, EUK-8 has been shown to significantly inhibit adipogenic differentiation in human adipose-derived stem cells by reducing ROS levels, thereby suppressing lipid accumulation [76]. This suggests EUK-8’s potential as a therapeutic agent for controlling fat formation and managing obesity-related conditions [76].

In a study, both EUK-8 and EUK-134 were found to significantly inhibit the aggregation of human islet amyloid polypeptide into amyloid fibrils, with EUK-134 showing slightly higher activity due to its additional ethoxy group [64,77]. This indicates their potential to protect pancreatic beta cells from amyloid-induced cytotoxicity in type 2 diabetes [64,77].

Similar to EUK-134, EUK-8 effectively reduced markers of liver damage and improved pathological features of NASH in rats, highlighting its potential as a therapeutic agent for preventing or treating NASH by mitigating oxidative stress [69].

Mn Salens, particularly the compounds EUK-134, EUK-207, and EUK-8, exhibit significant therapeutic potential across various conditions by mimicking SOD activity and managing oxidative stress. EUK-134 has shown effectiveness in conditions such as ischemia–reperfusion injury, breast cancer, muscle atrophy, and pulmonary hypertension by reducing oxidative stress and modulating key signaling pathways. EUK-207 has demonstrated protective effects against radiation-induced injuries, including lung damage, dermatitis, and cognitive impairments, highlighting its role as a radioprotective agent. Meanwhile, EUK-8 has proven beneficial in inhibiting adipogenic differentiation and amyloid aggregation, indicating its utility in managing obesity and type 2 diabetes. Overall, these findings highlight the wide-ranging potential of Mn Salens, especially in treating conditions where managing oxidative stress is crucial. The research findings of Mn Salens are summarized in Table 3.

### 2.3. MitoQ10

**Figure 2:**
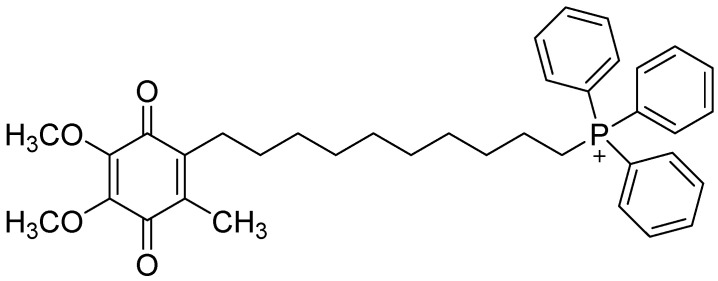
Structure of MitoQ10.

A molecule requires both a positive charge and lipophilicity for effective mitochondrial targeting. These characteristics were incorporated into the design of MitoQ10. MitoQ10’s structure features a redox-cycling quinone, an analog of mitochondrial ubiquinone, linked to a cationic triphenylphosphonium ion through a long lipophilic alkyl chain (Figure 2) [78]. The length of this alkyl chain directly influences the mitochondrial accumulation of MitoQ, with the optimized MitoQ10 molecule, containing a 10-carbon alkyl chain, showing the capacity to reduce mitochondrial oxidative stress [78]. Upon entering cells and mitochondria, MitoQ10 is rapidly reduced by the mitochondrial respiratory chain to its stable quinol form, MitoQH2, which is essential for its function as a reducing agent and antioxidant, with its major in vivo metabolite, monosulfonated MitoQ10, regenerating MitoQH2 after losing the sulfonate group [79]. MitoQ10’s forms a semiquinone radical (MitoQH^•^) which dismutates into MitoQ10 and MitoQH2, with MitoQH2, located in the membrane’s hydrophobic core, minimally reacting with superoxide but facilitating its dismutation through interactions with protonated superoxide (HO_2_^•^) and other reactive species [80]. MitoQ10 also reacts with peroxynitrite (ONOO^−^) to form MitoQH•, effectively preventing lipid peroxidation, influences cellular transcriptional activity by modulating signaling species, blocks H_2_O_2_-induced apoptosis and cell death, and its semiquinone form may act as a pro-oxidant, potentially triggering an adaptive response that upregulates endogenous antioxidant defenses, similar to Mn porphyrins [78,81,82,83].

As previously published in a review by Miriyala et al. in 2012, MitoQ10 had already been shown to function in addressing mitochondrial dysfunction through various analogs, including derivatives with redox-active moieties like vitamin E and dihydroethidium, and demonstrated therapeutic efficacy in animal models of oxidative stress, such as type I diabetes nephropathy, cardiac ischemia/reperfusion, and doxorubicin-induced cardiac toxicity, as well as in clinical trials for Parkinson’s disease and chronic liver hepatitis in hepatitis C virus-infected patients, although with mixed outcomes [26]. Since 2012, additional studies have provided further evidence supporting the therapeutic potential of MitoQ10 across various diseases by targeting mitochondrial dysfunction and oxidative stress [84,85,86,87,88,89,90,91]. In the context of cardiovascular diseases, MitoQ10 has shown promising effects. For instance, in patients with coronary artery disease and type 2 diabetes, mitochondrial ROS were found to elevate AMP-activated protein kinase (AMPK) activity, which plays a role in cellular defense against oxidative stress. However, despite this activation, endothelial function did not significantly improve, suggesting that while AMPK activation is a protective response, it may not be sufficient alone to reverse endothelial dysfunction in these conditions [84]. Additionally, the combination of MitoQ10 and the angiotensin receptor blocker losartan was found to significantly reduce blood pressure, left ventricular hypertrophy, and cardiac fibrosis in hypertensive models, highlighting MitoQ10’s potential in treating resistant hypertension and preventing related end-organ damage [85]. Furthermore, MitoQ10’s ability to reduce mitochondrial oxidative damage was corroborated in studies showing its antihypertrophic effects on cardiomyocytes, indicating its broader therapeutic relevance in cardiovascular disease management [85].

In neurodegenerative conditions, MitoQ10 has been explored for its neuroprotective effects. It was demonstrated to prevent both caspase-dependent and caspase-independent neuronal death by blocking mitochondrial ROS production and preserving mitochondrial function in models of neurotrophin deficiency [86]. This indicates that MitoQ10 may offer a promising therapeutic approach for diseases where mitochondrial dysfunction contributes to neuronal cell death [86]. In renal dysfunction associated with hypertension, MitoQ10 mirrored the protective effects of chronic aerobic exercise by preserving mitochondrial function, enhancing ATP production, and reducing oxidative stress, further underlining its potential in mitigating renal damage through mitochondrial protection [87].

MitoQ10’s application extends beyond cardiovascular and neurodegenerative diseases into other areas, such as cancer and metabolic disorders. In cancer research, MitoQ10, along with curcumin, was studied for its role in promoting the crosstalk between autophagy and apoptosis, particularly through mitochondrial destabilization [88]. This finding suggests potential therapeutic strategies that leverage MitoQ10’s capacity to modulate these cellular processes in cancer treatment [88]. In polycystic ovary syndrome (PCOS), MitoQ10 showed significant potential in improving insulin resistance and reducing oxidative stress, demonstrating its ability to reverse endocrine and reproductive abnormalities, including in models where PCOS was induced by circadian rhythm disruption [89,90]. Moreover, MitoQ10’s impact on mitochondrial function was also beneficial in cryopreservation contexts, where it enhanced post-thaw sperm quality and antioxidant status, although dose optimization was necessary to avoid adverse effects [91].

Overall, MitoQ10 has demonstrated a wide range of therapeutic applications across various diseases by targeting mitochondrial dysfunction and oxidative stress. Its benefits in cardiovascular, neurodegenerative, metabolic, and reproductive health, as well as in cancer therapy, underscore its potential as a versatile mitochondria-targeted antioxidant. However, careful consideration of dosage and treatment context remains crucial to maximize its therapeutic efficacy while minimizing potential adverse effects.

### 2.4. Nitroxides

**Figure 3:**
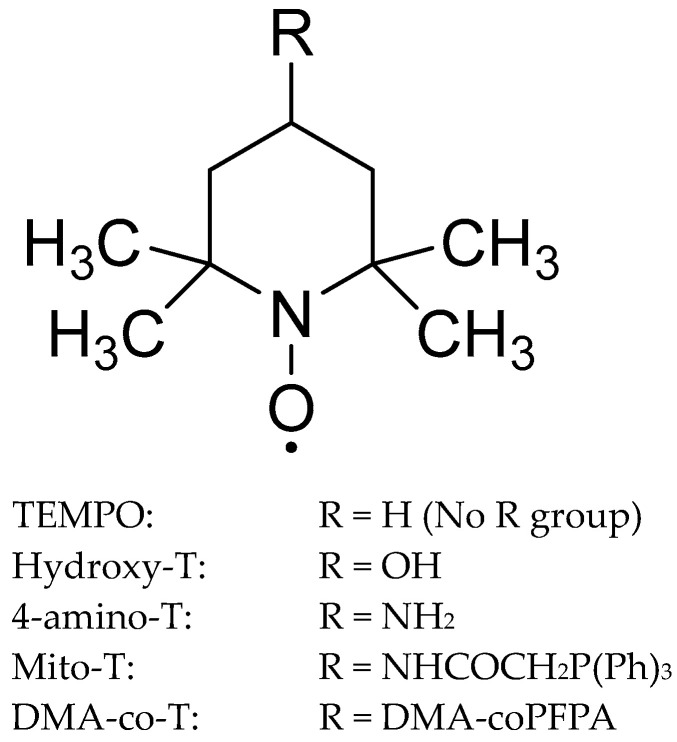
Core structure of TEMPO compounds. For TEMPO compounds with longer names, T was used as an abbreviation for TEMPO. DMA-coPFPA is a copolymer made from dimethylacrylamide (DMA) and pentafluorophenyl acrylate (PFPA), often used to enhance targeted delivery.

Nitroxides are a class of stable free radicals that have been explored as MnSOD mimetics due to their ability to scavenge superoxide and other ROS. While they do not contain manganese, nitroxides can mimic the activity of MnSOD by catalytically dismutating O_2_^•−^. Tempo (2,2,6,6-tetramethylpiperidine-N-oxyl) is a stable free radical, and while not considered a MnSOD mimetic due to its lack of functional group capable of superoxide scavenging, it can function as an antioxidant by scavenging free radicals. It is characterized by a six-membered piperidine ring with four methyl groups attached to it, creating a bulky, sterically hindered structure. The presence of a nitroxyl group (-NO^•^) on the piperidine ring is what gives tempo its free radical properties. As nitroxides of interest are derived from tempo, tempo will also be reviewed (Figure 3).

Tempo has been explored in multiple studies for its ROS-scavenging abilities. It effectively reversed the inhibitory effects of hypoxia/reoxygenation on acetylcholine-induced vasodilation in cerebral arteries, highlighting its role in protecting against oxidative stress-induced endothelial dysfunction [92]. Additionally, Tempo was found to significantly reduce cyclooxygenase-2 expression under hypertonic stress in collecting duct cells by mitigating mitochondrial-derived ROS, underscoring its potential in protecting renal cells from osmotic stress-induced damage [93]. Furthermore, Tempo has shown efficacy in reducing angiotensin II (ANG II)-induced calcium signaling in vascular smooth muscle cells by scavenging superoxide anions, suggesting its utility in preventing ROS-mediated vascular complications [94].

Hydroxy-TEMPO (also known as Tempol or 4-Hydroxy TEMPO) has been extensively studied for its protective role in various oxidative stress-related conditions [94,95,96,97]. In pulmonary vascular remodeling induced by chronic hypoxia, Hydroxy-TEMPO mitigated pulmonary damage by reducing ROS levels and normalizing media wall thickness of pulmonary arteries, highlighting its therapeutic potential in pulmonary vascular conditions [95]. In vascular studies, Hydroxy-TEMPO inhibited ANG II-induced increases in intracellular calcium concentration in afferent arterioles, underscoring its role in modulating ROS-related vascular functions [94]. Additionally, it has been employed as a superoxide detection probe in studies on endothelin receptor-mediated calcium signaling, supporting its utility in studying oxidative stress modulation in vascular systems [96]. Furthermore, Hydroxy-TEMPO protected microglial cells against neurotoxic effects from dichlorvos (an organophosphate pesticide) by effectively reducing ROS and nitric oxide (NO) production, downregulating inflammatory markers, and preventing apoptosis, thus indicating its potential as a therapeutic agent for neurodegenerative disorders characterized by oxidative stress and inflammation [97].

4-amino-Tempo (AT), a derivative of Tempo, has been investigated for its antihypertensive effects in spontaneously hypertensive rats [98]. AT, along with Tempol, was shown to significantly reduce mean arterial pressure and heart rate, with these effects closely linked to their SOD mimetic activity [98]. This finding indicates that 4-amino-Tempo could be a promising candidate for managing hypertensive crises due to its potent blood pressure-lowering effects [98].

TEMPO-9-AC was employed as a fluorescent probe in vascular studies, particularly in the context of endothelin receptor-mediated signaling [96]. It helped demonstrate the role of superoxide in enhancing calcium signaling through the adenosine diphosphate ribose cyclase pathway, providing insight into the molecular mechanisms underlying ROS-mediated vascular dysfunctions [96].

Mito-TEMPO, a mitochondria-targeted SOD mimetic, has been investigated in various models of oxidative stress [99,100,101]. It significantly inhibited the NOD-like receptor family pyrin domain containing 3 inflammasome activation and reduced mitochondrial ROS production in influenza virus-infected macrophages, suggesting its potential in mitigating viral-induced lung injury [99]. Mito-TEMPO also demonstrated protective effects against sodium fluoride-induced cytotoxicity in embryonic carcinoma cells by enhancing Sirt1 expression and promoting the deacetylation of the human MnSOD gene, thereby reducing mitochondrial oxidative damage [100]. Additionally, Mito-TEMPO was effective in reducing ROS levels and oxidative stress in models of hypertension and vascular dysfunction, highlighting its utility in targeting mitochondrial ROS to prevent cardiovascular complications [101].

Poly(DMA-co-TEMPO) copolymers were developed to optimize the antioxidant and anti-inflammatory effects of TEMPO [102]. These copolymers demonstrated superior retention and efficacy in reducing ROS levels and inflammation in vivo compared to free TEMPO, suggesting their potential in treating inflammatory diseases driven by oxidative stress [102].

In summary, nitroxides, derived from the stable free radical TEMPO, have proven to be versatile agents in mitigating oxidative stress across a variety of biological contexts. From protecting pulmonary and vascular structures against ROS-induced damage to demonstrating antihypertensive effects and safeguarding against neurotoxic insults, TEMPO’s derivatives have consistently shown potential as MnSOD mimetics. Their application spans from basic research, such as the study of membrane protein interactions, to potential therapeutic roles in conditions involving oxidative stress, inflammation, and mitochondrial dysfunction. The broad spectrum of efficacy observed with hydroxy-TEMPO, 4-amino-Tempo, and Mito-TEMPO underscores their value in developing novel strategies for managing diseases where oxidative damage plays a central role. As research progresses, the unique properties of these compounds continue to offer promising avenues for therapeutic development, particularly in addressing the complex interplay of ROS in various pathological processes. 

### 2.5. Mangafodipir

Although mangafodipir was removed from the Drug Product List by the FDA in 2003, and withdrawn from the European market in 2012, it and its derivative calmangafodipir have garnered significant attention due to their potential to mitigate oxidative stress and enhance the therapeutic index of various treatments, particularly in the context of chemotherapy and oxidative stress-related conditions. Mangafodipir is currently exlusively used in research.

**Figure 4:**
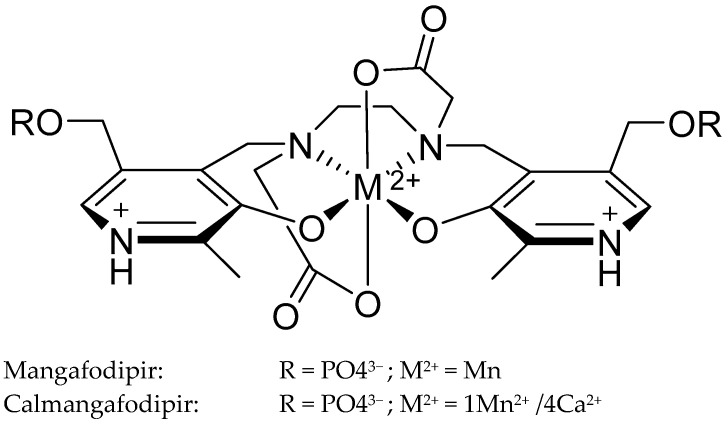
Structure of Mangafodipir and Calmangafodipir [103].

Mangafodipir is a complex compound where manganese is chelated with the ligand fodipir, forming a stable chelate that mimics SOD activity (Figure 4). This SOD mimetic activity allows mangafodipir to catalyze the dismutation of O_2_^•−^ into O_2_ and H_2_O_2_, thereby reducing oxidative stress [103]. It has demonstrated protective effects against acetaminophen-induced liver injury, chemotherapy-induced peripheral neuropathy (CIPN), and ischemia–reperfusion injury, among other conditions [104,105,106,107,108,109,110]. Notably, Mangafodipir has shown efficacy in protecting against oxidative damage in various preclinical models, including reducing ROS in hepatocytes and preserving mitochondrial function in the liver during ischemia–reperfusion injury [107]. Additionally, it has been reported to enhance the therapeutic effects of chemotherapy by reducing hematologic toxicity while simultaneously increasing the cytotoxicity against cancer cells, thereby improving the overall therapeutic index of chemotherapeutic regimens [105,110].

Calmangafodipir is a derivative of mangafodipir, designed to improve the stability and safety profile by reducing manganese toxicity. Calmangafodipir achieves this by replacing a significant portion of the manganese with calcium, thereby doubling the renal excretion of Mn^2+^ and minimizing its retention in critical organs such as the brain and pancreas [103,111]. This modification not only enhances the stability of the compound but also increases its therapeutic efficacy [112]. Calmangafodipir has been explored extensively for its potential to prevent CIPN, particularly in patients treated with oxaliplatin, a chemotherapy drug known for its neurotoxic effects. Preclinical studies have shown that calmangafodipir effectively reduces oxidative stress, protects neurons from damage, and preserves nerve function, thereby preventing CIPN [112]. However, the results from the prevention of oxaliplatin-induced peripheral neuropathy phase III trials, which aimed to evaluate its efficacy in preventing CIPN in colorectal cancer patients, were unexpectedly negative [113]. The trials reported increased CIPN incidence in patients treated with calmangafodipir, which was attributed to unfavorable redox interactions between manganese in calmangafodipir and platinum in oxaliplatin [113]. These interactions likely exacerbated oxidative stress, leading to increased nerve damage, ultimately resulting in the discontinuation of the trials [113,114,115,116,117].

Despite these challenges, both mangafodipir and calmangafodipir continue to be of interest in various therapeutic contexts. Calmangafodipir has shown potential as an adjunct therapy in treating acetaminophen overdose, particularly by reducing oxidative stress and mitigating mitochondrial damage, though its efficacy in clinical outcomes remains inconclusive. Furthermore, Mangafodipir has been proposed as a supplementary treatment in conditions like COVID-19 to reduce endothelial inflammation and thrombosis; however, more research is needed to fully establish its clinical benefits [116].

## 3. Conclusions

The exploration of MnSOD mimetics has significantly advanced our understanding and potential management of oxidative stress-related conditions. Controlled redox reactions are integral to vital biological processes, and the disruption of redox balance leads to the overaccumulation of ROS, causing extensive cellular damage. MnSOD, located in the mitochondrial matrix, is pivotal in neutralizing O_2_^•−^, especially in tissues with high metabolic demand.

Historically, the development of MnSOD mimetics has transitioned from general SOD mimics to compounds specifically emulating MnSOD’s catalytic mechanisms. Among these, Mn porphyrins have been extensively studied due to their ability to localize within mitochondria and modulate cellular redox balance. Variants such as MnTBAP, MnTE, MnTnHex, and MnTnBuOE have shown promise in therapeutic applications ranging from reproductive biology to cancer therapy and radioprotection.

Mn salens, including EUK-134, EUK-207, and EUK-8, have demonstrated significant potential as SOD and catalase mimetics. Their efficacy in reducing oxidative stress has implications for treating conditions such as ischemia–reperfusion injury, muscle atrophy, pulmonary hypertension, and radiation-induced injuries. These compounds have shown the ability to modulate key signaling pathways and protect against oxidative damage in various tissues.

MitoQ10, a mitochondria-targeted antioxidant, has shown versatility in addressing mitochondrial dysfunction across cardiovascular, neurodegenerative, metabolic, and reproductive disorders. Its ability to reduce mitochondrial oxidative damage underscores its potential as a therapeutic agent in diseases where mitochondrial ROS play a central role. By modulating cellular signaling and protecting mitochondrial function, MitoQ10 offers a promising approach to mitigating oxidative stress-related cellular damage.

Nitroxides, such as TEMPO and its derivatives, offer a different approach by catalytically dismutating O_2_^•−^ without containing manganese. Their broad efficacy in mitigating oxidative stress-related damage in pulmonary, vascular, neurodegenerative, and inflammatory conditions highlights their value in therapeutic development. Mito-TEMPO, in particular, has shown effectiveness in targeting mitochondrial ROS, offering protective effects in models of hypertension and vascular dysfunction.

Mangafodipir and its derivative calmangafodipir have also contributed to the field by enhancing the therapeutic index of treatments, particularly in CIPN and oxidative stress-related conditions. Despite challenges faced in clinical trials, they continue to be of interest due to their SOD mimetic activity and potential to mitigate treatment-related side effects.

These MnSOD mimetics represent a promising frontier in treating oxidative stress-related diseases, offering therapeutic potential by mimicking the body’s natural antioxidant defenses. While all these compounds effectively dismute O_2_^•−^, optimizing their stability and bioavailability, as well as minimizing potential toxicity through R-group modifications can further enhance their efficacy. Although some MnSOD mimetics benefit both healthy and diseased tissues in certain conditions, such as adjuvant chemotherapy and radiotherapy, advancing tissue-selective and mitochondrial targeting could broaden their therapeutic impact. Future research should explore MnSOD mimetics not only as treatments for diseases linked to oxidative stress but also as preventive adjuvants where disease progression or standard therapies are expected to elevate ROS levels and cause mitochondrial damage.

## Figures and Tables

**Table 1:** MnSOD fast and slow reactions [22].

Equation #	Simple Equation	Full Equation	Rate Constant
(1)	EA + O_2_^•−^ → EB + O_2_	Mn^3^⁺SOD(OH)^−^ + O_2_^•−^ → Mn^2^⁺SOD(H_2_O) + O_2_	k_1_
(2)	EB + O_2_^•−^ + 2H⁺ → EA + H_2_O_2_	Mn^2^⁺SOD(H_2_O) + O_2_^•−^ → Mn^3^⁺SOD(OH)^−^ + H_2_O_2_	k_2_
(3)	EB + O_2_^•−^ → EC	Mn^2^⁺SOD(H_2_O) + O_2_^•−^ → Mn^3^⁺SOD(H_2_O)(OO^2−^)	k_3_
(4)	EC → EA	Mn^3^⁺SOD(H_2_O)(OO^2−^) → Mn^3^⁺SOD(OH)^−^ + H_2_O_2_	k_4_

**Table 2:** Summary of Research Implications of Mn Porphyrins.

Mimetic	Research Area	Finding Summary
MnTBAP	Reproductive Health	In horse and pig sperm cryopreservation MnTBAP enhanced membrane integrity, motility, and viability, while reducing reactive oxigen species (ROS) and apoptosis, improving embryo development in pigs [33,34]. In human sperm cryopreservation, it decreased ROS without impacting motility at moderate concentrations [35,36].
	Cardiovascular Health	In rats with pulmonary hypertension, it improved vascular remodeling, cardiac function, and oxidative stress markers by modulating bone morphogenetic protein receptor type 2 levels and autophagy [37]. In Sirtuin2-deficient mice, MnTBAP reduced mitochondrial ROS, vascular stiffness, and aging-related remodeling [38].
	Chemotherapy Induced Injury	In mice with cisplatin-induced kidney injury and cochlear synaptopathy, MnTBAP lowered oxidative stress, lipid peroxidation, and apoptosis, improved kidney function, and preserved auditory responses [39,40].
MnTM-2-PyP5+ (MnTM)	Renal Health	Late administration of MnTM exacerbated diabetic complications, particularly kidney damage, due to pro-oxidative effects rather than the anticipated antioxidant protection [41].
MnTE-2-PyP5+ (MnTE)	Radiation Induced Injury	In rat models of radiation proctitis, MnTE prevented acute and chronic symptoms with greater efficacy when administered as a pre-treatment [42].
	Diabetes	In mouse models of type 1 diabetes, it modulated immune cell metabolism, promoting metabolic quiescence and delaying diabetes onset [43].
	Cardiovascular Disease	During myocardial ischemia/reperfusion injury, co-administration with S-nitrosoglutathione improved outcomes in non-diabetic mice while mitigating worsened outcomes in diabetic mice by reducing peroxynitrite production [44].
	Radiotherapy	In breast cancer, MnTE enhanced tumor growth suppression during radiation therapy [45].
	Sickle Cell Disease	For sickle cell disease, MnTE reduced red blood cell and leukocyte adhesion, lowered ROS levels, restored blood flow, and improved survival in sickle cell mice [46].
MnTnHex-2-PyP5+ (MnTnHex)	Radiotherapy	In breast cancer, MnTnHex enhanced tumor suppression when combined with radiation therapy [45]. In mouse cancer models, MnTnHex enhanced radiosensitivity by promoting apoptosis, increasing ROS, and suppressing DNA repair and prosurvival signaling [47].
	Chemotherapy	For non-small cell lung cancer and clear-cell renal carcinoma, MnTnHex showed concentration-dependent cytotoxicity, reducing cell viability, promoting apoptosis, and enhancing effects when combined with cisplatin [48,49].
	Radiation Induced Injury	For pulmonary radioprotection, it significantly reduced radiation-induced lung damage, oxidative stress, and inflammation in rats and mice [50,51]. In nonhuman primates, it delayed pneumonitis onset, reduced respiratory distress, and mitigated fibrosis-related lung weight increases [52].
MnBuOE-2-PyP5+ (MnBuOE)	Sickle Cell Disease	MnBuOE exhibited protective and therapeutic effects across multiple conditions. In sickle cell disease, it reversed red blood cell and leukocyte adhesion, reduced ROS, restored blood flow, and improved survival in mice [46].
	Radiation Induced Injury	In models of cranial irradiation, MnBuOE preserved hippocampal neurogenesis and enhanced neuron survival, indicating potential as a brain radioprotector [53].
	Chemotherapy	In cancer, it increased oxidative stress selectively in tumor cells, enhancing apoptosis in glioblastoma, ovarian, and lung cancer cells, especially when combined with treatments like tumor necrosis factor-related apoptosis-inducing ligand, carboplatin, and cisplatin [54,55,56].
	Radiotherapy	MnBuOE also showed enhanced tumor suppression in combination with radiotherapy, promoting apoptosis through redox modulation and H_2_O_2_ production [57].
	Cardiovascular Disease	In cardiovascular applications, MnBuOE improved recovery post-cardiac arrest by reducing neuronal and kidney injury, and it lowered blood pressure in hypertensive models by reducing sympathetic nerve activity, scavenging ROS, and inducing vasodilation [58,59]
HSJ-0017	Radiotherapy/ Chemotherapy	HSJ-0017 significantly inhibited superoxide generation and scavenged H_2_O_2_ [60]. HSJ-0017 enhanced antitumor effects in sarcoma 180 tumor-bearing mice but not in hepatocarcinoma 22 tumor xenografts [60]. It also reduced the toxicities associated with radiotherapy and chemotherapy while exhibiting anti-inflammatory and hepatoprotective effects [60].

**Table 3:** Summary of research findings of Mn Salens.

Mimetic	Research Area	Findings
EUK-134	Pulmonary Disease	In mouse models, EUK-134 reduced hyperoxia-induced fibrosis by selectively modulating transforming growth factor-beta 1 signaling [63]. In pulmonary hypertension-induced diaphragm dysfunction, EUK-134 preserved muscle contractility and countered oxidative damage [71].
	Cancer	In human breast cancer cells, EUK-134 inhibited ROS production, cell proliferation, and migration, while inducing apoptosis [64]. EUK-134 also inhibited nuclear factor kappa-light-chain-enhancer of activated B cells activation and expression of metastatic factors in cancer models [66].
	Hematological Disorders	EUK-134 suppressed ROS-mediated platelet activation in response to thromboxane analogs [67].
	Mitochondrial Stress	In ER stress-induced mitochondrial dysfunction, EUK-134 improved mitochondrial health by reducing ROS and enhancing respiration [68].
	Liver Disease	In liver disease models, EUK-134 reduced oxidative stress and pathological changes in non-alcoholic steatohepatitis (NASH) and prevented progression from non-alcoholic fatty liver disease to NASH [69,70].
	Muscular Disorders	EUK-134 reduced oxidative stress and preserved muscle integrity during mechanical unloading in rats, preventing muscle atrophy and maintaining nNOS localization [65].
EUK-207	Neurological Disorders	EUK-207 showed neuroprotective effects in traumatic brain injury by reducing oxidative stress, limiting neuronal death, enhancing cognitive function, and suppressing acute and chronic neuroinflammatory responses [72]. EUK-207 mitigated radiation-induced cognitive deficits in mice, reducing oxidative stress markers in the brain without affecting non-irradiated controls [75].
	Radiation Induced Injury	In radiation-induced lung damage, EUK-207 decreased lung fibrosis, oxidative DNA damage, and inflammation markers, with combined therapy (EUK-207 and captopril) providing superior protection [73]. For radiation dermatitis, EUK-207 improved wound healing by reducing oxidative stress, normalizing gene expression, and promoting angiogenesis [74].
EUK-8	Metabolic Disorders	EUK-8 suppressed ROS production and reduced adipogenic differentiation and lipid accumulation in human adipose-derived stem cells during adipogenesis, decreasing both differentiated cell proportion and intracellular lipid levels [76].
	Neurological Disorders	In combination with EUK-134, EUK-8 inhibited amyloid formation in human islet amyloid polypeptide, reducing cytotoxicity in SK-N-MC cells by enhancing cell viability and reducing lactate dehydrogenase release [77].
